# Scavenging patterns of an inbred wolf population in a landscape with a pulse of human‐provided carrion

**DOI:** 10.1002/ece3.10236

**Published:** 2023-07-04

**Authors:** Camilla Wikenros, Cecilia Di Bernardi, Barbara Zimmermann, Mikael Åkesson, Maike Demski, Øystein Flagstad, Jenny Mattisson, Aimee Tallian, Petter Wabakken, Håkan Sand

**Affiliations:** ^1^ Grimsö Wildlife Research Station, Department of Ecology Swedish University of Agricultural Sciences Riddarhyttan Sweden; ^2^ Department of Biology and Biotechnologies “Charles Darwin” University of Rome La Sapienza Rome Italy; ^3^ Faculty of Applied Ecology, Agricultural Sciences and Biotechnology Inland Norway University of Applied Sciences Elverum Norway; ^4^ County Administrative Board of Norrbotten Luleå Sweden; ^5^ Norwegian Institute for Nature Research (NINA) Trondheim Norway

**Keywords:** *Canis lupus*, consumption time, human density, inbreeding, intraguild competition, prey density, social affiliation

## Abstract

Scavenging is an important part of food acquisition for many carnivore species that switch between scavenging and predation. In landscapes with anthropogenic impact, humans provide food that scavenging species can utilize. We quantified the magnitude of killing versus scavenging by gray wolves (*Canis lupus*) in Scandinavia where humans impact the ecosystem through hunter harvest, land use practices, and infrastructure. We investigated the cause of death of different animals utilized by wolves, and examined how the proportion of their consumption time spent scavenging was influenced by season, wolf social affiliation, level of inbreeding, density of moose (*Alces alces*) as their main prey, density of brown bear (*Ursus arctos*) as an intraguild competitor, and human density. We used data from 39 GPS‐collared wolves covering 3198 study days (2001–2019), including 14,205 feeding locations within space–time clusters, and 1362 carcasses utilized by wolves. Most carcasses were wolf‐killed (80.5%) while a small part had died from other natural causes (1.9%). The remaining had either anthropogenic mortality causes (4.7%), or the cause of death was unknown (12.9%). Time spent scavenging was higher during winter than during summer and autumn. Solitary wolves spent more time scavenging than pack‐living individuals, likely because individual hunting success is lower than pack success. Scavenging time increased with the mean inbreeding coefficient of the adult wolves, possibly indicating that more inbred individuals resort to scavenging, which requires less body strength. There was weak evidence for competition between wolves and brown bears as well as a positive relationship between human density and time spent scavenging. This study shows how both intrinsic and extrinsic factors drive wolf scavenging behavior, and that despite a high level of inbreeding and access to carrion of anthropogenic origin, wolves mainly utilized their own kills.

## Graphical abstract

The major part of wolves' consumption time was spent on fresh wolf‐killed ungulates, and carcasses due to livestock depredation, intraguild predation, and intraspecific killing were rare. Scavenging only accounted for a small portion of the total consumption and was more common in winter and among solitary wolves. Inbreeding was related to the level of scavenging; the more inbred the wolves were, the more they scavenged.

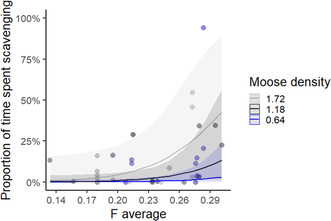

## 1 INTRODUCTION

Carnivores acquire food via predation (i.e., killing prey) and/or via scavenging (i.e., opportunistically utilizing carrion) (Schaller, 1972). The level of predation versus scavenging varies between species, populations, and individuals, and can change in response to intrinsic and extrinsic factors (Pereira et al., 2014). Carnivores can switch to scavenging during periods when prey are less vulnerable to predation (Pereira et al., 2014), when the density of accessible prey is low (Messier & Crete, 1985; Tallian, Smith, et al., 2017), or when anthropogenic food sources are readily available (Mattisson et al., 2016). Individual body size of carnivores can also affect levels of scavenging, as body size plays a key role in hunting success (MacNulty, Smith, Mech, et al., 2009). However, most carnivores commonly scavenge when encountering a carcass (DeVault et al., 2003; Selva et al., 2005; Wilson & Wolkovich, 2011), and scavenging is therefore an important part of food acquisition for many carnivore species.

For large carnivores, the level of scavenging versus predation can differ between anthropogenic landscapes and protected areas. Carrion provided by humans can also be preferred, especially when the accessibility and abundance of wild prey is low (Newsome et al., 2015). For top predators such as gray wolves (*Canis lupus*), diet can be altered with the access to anthropogenic food sources like livestock (e.g., via depredation), carcass dumps, and garbage sites (Newsome et al., 2015). For example, depredation was common by wolves in Portugal (Vos, 2000), the majority of scavenging done by wolves in Italy constituted livestock carrion (Ciucci et al., 2020), and wolves utilized garbage in southern Europe (Zlatanova et al., 2014).

The provision of anthropogenic food sources can show large variation in time (Wikenros et al., 2013). For example, the pulse of slaughter remains during the moose (*Alces alces*) hunting season in Scandinavia is utilized by an array of carnivore species (Gomo et al., 2017; Wikenros et al., 2013). Human activities not only result in a direct provision of food sources in terms of carrion but can also affect carnivores' access to wild prey. Due to intensive moose harvest in Scandinavia, the body condition of surviving moose is generally high (Sand et al., 2012) and the rate of non‐harvest mortality low (Broman et al., 2002; Ericsson et al., 2001; Rönnegård et al., 2008). As a consequence, less biomass is available for scavengers from moose dying of causes other than hunter harvest, for example, winter die‐off (Wikenros et al., 2013).

In this study, we explore patterns of scavenging and predation in an anthropogenic landscape in Scandinavia using data from 82 study periods, where we searched for carcasses utilized by GPS‐collared wolves, performed between 2001 and 2019. First, we classified the cause of death of carcasses utilized by wolves and estimated the proportion of consumption time spent at scavenged carrion versus wolf‐kills. Second, we examined how the proportion of consumption time spent scavenging (hereafter scavenging time) by wolves was affected by a set of intrinsic and extrinsic factors shown, or hypothesized, to be of importance for wolf feeding patterns.

The Scandinavian wolf population has been subject to loss of genetic diversity, and high levels of inbreeding, since the current population was founded in 1983 (Åkesson, Flagstad, et al., 2022; Vilà et al., 2003; Viluma et al., 2022). This has caused both negative effects on individual fitness (Åkesson et al., 2016; Liberg et al., 2005; Milleret et al., 2017; Wikenros et al., 2021) and increasing incidence of congenital anomalies (Räikkönen et al., 2006). Inbreeding has been shown to negatively affect body condition in several wolf populations (Fredrickson & Hedrick, 2002; Keller & Waller, 2002; Laikre & Ryman, 1991). Highly inbred wolves might therefore be less successful when hunting large ungulate prey and thus more likely to resort to scavenging, which requires less body strength. We predicted that scavenging time would be greater with higher inbreeding due to an associated decrease in body condition. We also predicted an increased scavenging time for solitary wolves, as previously observed (Bassi et al., 2018). Solitary wolves are commonly younger and less experienced hunters, and are expected to have reduced hunting efficiency, compared to pack‐living individuals (MacNulty et al., 2014; Sand et al., 2006a; Zimmermann et al., 2015).

Season may also affect the level of scavenging exhibited by wolves given that moose natural mortality occurs during late winter while there is a greater availability of carrion with anthropogenic origin during autumn due to the moose hunt (Wikenros et al., 2013). Thus, we predicted that scavenging time would be highest during the autumn hunting season. We also predicted that scavenging time would increase as moose density declined, as it becomes more difficult to find vulnerable individuals in accordance with a predicted functional response (Zimmermann et al., 2015), and with increased brown bear (*Ursus arctos*) density. When sympatric with brown bears, wolf kill rate decreases as a result of interference competition during spring and exploitation competition during summer (Tallian et al., 2022; Tallian, Ordiz, et al., 2017). Thus, wolves living with bears may scavenge more often to make up for food lost via kleptoparasitism. We also explored the effect of anthropogenic impact on wolf foraging patterns by testing for an effect of human density on time spent scavenging. We predicted an increased scavenging time with higher human density as it likely results in a greater presence of food sources with anthropogenic origin (Oro et al., 2013) such as remains from hunter‐kills, vehicle collisions, bait stations, and illegal dumping of livestock carrion.

Our study provides a detailed documentation of the feeding ecology of an inbred wolf population inhabiting a landscape with intensive management of ungulates and large carnivores. The results have implications for the management of ungulate population as the predation/scavenging ratio will influence the impact of wolves on prey population growth. In addition, the study provides knowledge of how wolves may impact co‐occurring species through their extent of utilization of carrion. This study also provides novel insight into the effects of inbreeding on the patterns of wolf feeding ecology.

## 2 METHODS

### 2.1 Study area

**FIGURE 1:**
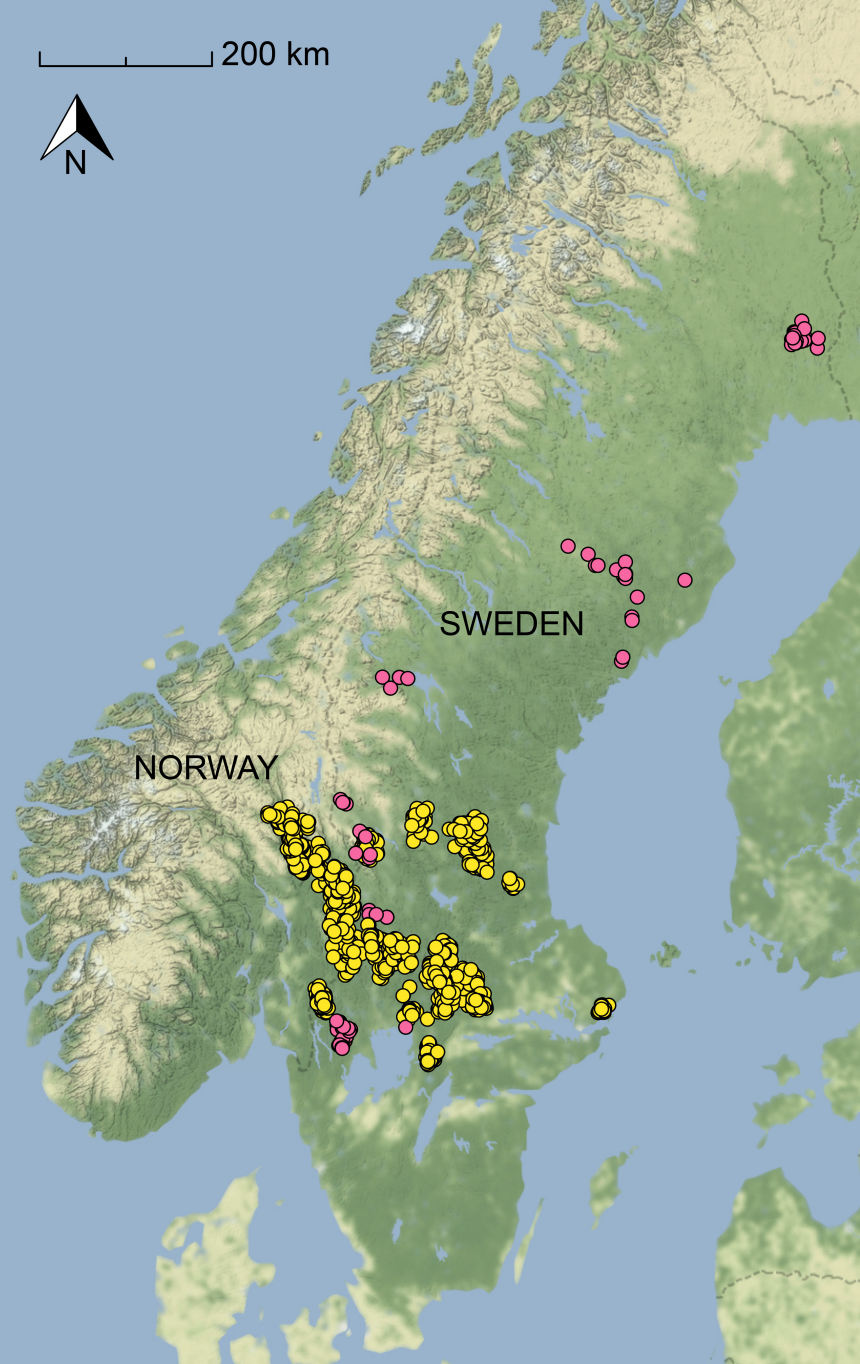
Sites with carcasses (*n* = 1362) found during intensive predation studies of solitary wolves (pink dots, *n* = 11) and adult wolves in packs (≥2 wolves, yellow dots, *n* = 71) in Scandinavia, 2001–2019.

The study was conducted in Scandinavia (Norway and Sweden) within the distribution range of the wolf population (Figure 1). The area mainly consisted of boreal forest, where most of the forest (composed of Norway spruce [*Picea abies*], Scots pine [*Pinus sylvestris*] and some deciduous tree species) was managed by clear‐cutting followed by regeneration, resulting in a mosaic of conifer stands in different age classes as well as an extensive network of forest roads. The climate was continental, and snow covered the ground mainly during December to March. Human density averaged 25 humans/km^2^ in Sweden and 15/km^2^ in Norway in 2020 (https://www.fn.no), with a mean of 9/km^2^ (range 1–79) within the wolf territories included in this study.

Wolves were extirpated from most of Scandinavia, including our study area, by the end of the 19th century and were functionally extinct by the 1960s. They returned to the study area in the late 1970s and early 1980s through natural re‐colonization from the Finnish/Russian wolf population and the first confirmed reproduction occurred in 1983 (Åkesson, Flagstad, et al., 2022). By the winter of 2019/2020, the population consisted of 71 territories, including 26 non‐reproducing and scent‐marking pairs and 45 family groups (≥3 wolves of which ≥1 was a scent‐marking adult wolf), with the majority (78%) of the territories located in Sweden (Wabakken et al., 2020). Mean family group size was 5.6 individuals (95% CI = 4.6–6.7; Chapron et al., 2016) and the largest documented family group during winter was 12 individuals (Svensson et al., 2021). The wolf population in Scandinavia is managed by lethal control aiming to regulate population size and reduce the socio‐economic impact of wolves on the local communities. However, no lethal control occurred during the intensive study periods in the wolf territories included in the study.

Moose are the main prey of wolves in Scandinavia (Sand et al., 2008; Zimmermann et al., 2015). The Scandinavian moose population has been one of the most heavily harvested ungulate populations in the world, with 25%–30% of the moose population harvested annually (Lavsund et al., 2003). The moose harvest season in Norway starts on September 25 and lasts until December 23. The harvest season in Sweden is allowed during 3 weeks in September and/or from the second Monday in October until the last day of January or February. Mean winter moose density was 1.3/km^2^ inside wolf territories (Zimmermann et al., 2015). Roe deer (*Capreolus capreolus*) density, an alternative prey for wolves, was generally below 0.5/km^2^ within wolf territories located in the central and northern part of the wolf breeding range, but reached up to 4.5/km^2^ in the more southern wolf territories (Sand et al., 2016).

Other large and medium‐sized carnivores in the study area included brown bear, Eurasian lynx (*Lynx lynx*), and wolverine (*Gulo gulo*). The most common scavenging species included red fox (*Vulpes vulpes*), common raven (*Corvus corax*), Eurasian jay (*Garrulus glandarius*), European pine marten (*Martes martes*), golden eagle (*Aquila chrysaetos*), and hooded crow (*Corvus cornix*) (Wikenros et al., 2013).

### 2.2 Wolf individual characteristics

Wolf social affiliation was classified as either solitary or pack (a scent‐marking pair or family group), based on the Scandinavian wolf monitoring system. Monitoring is conducted annually from October 1 to March 31, mainly using snow tracking combined with DNA analysis of scats and urine (Åkesson, Svensson, et al., 2022).

Based on a reconstructed pedigree of confirmed breeding pairs since the current Scandinavian wolf population was founded (Åkesson et al., 2016; Liberg et al., 2005), the inbreeding coefficient (*F*) of adult breeding females and males in packs (solitary wolves were excluded from the analyses including the effect of inbreeding) was calculated using CFC v. 1.0 (Sargolzaei et al., 2005). The adult pair within a pack usually move together and are primarily responsible for the hunting of ungulates among pack members (Nordli et al., 2023; Zimmermann et al., 2015). We therefore tested the average inbreeding coefficients of the adult female and male in a pack (*F*_average_) in the analyses, as well as the inbreeding coefficient of the adult male (*F*_male_) given the greater body size of adult males compared to adult females which may imply a greater contribution to the hunting success (MacNulty, Smith, Mech, et al., 2009; Sand et al., 2006a).

### 2.3 Intensive studies of predation

To identify carcasses utilized by wolves, we used GPS‐data collected from collared wolves (GPS‐Simplex or Tellus TVP Positioning/Followit and GPS‐Plus Vectronic Aerospace), stored in a Wireless Remote Animal Monitoring database system for data validation and management (Dettki et al., 2014). All procedures including capture, handling, and collaring of wolves (Sand et al., 2006a) fulfilled ethical requirements and were approved by the Swedish Animal Welfare Agency and the Norwegian Experimental Animal Ethics Committee.

A total of 82 intensive studies were conducted between 2001 and 2019 on 39 wolf individuals, 34 in summer (15 May to 14 September, 26 collared wolves), eight in autumn (15 September to 14 December, six collared wolves), and 40 in winter (15 December to 14 May, 28 collared wolves). Definition of the different seasons was based on variation in carcass availability (Wikenros et al., 2013). Seven studies overlapped two seasons by 1–8 days (median 2 days). These were assigned to the season including the majority of the study period. Eleven of the studies were on solitary wolves (*n* = 7 individuals), while 71 were on packs (*n* = 32 individuals). We only used data from one of the adult wolves in the same pack (*n*_males_ = 61, *n*_females_ = 10). Solitary wolves were either individuals captured and collared as pups in their natal territory during their first winter and where intensive studies were conducted during the dispersal phase (*n* = 2) or after established in a new territory (*n* = 3), or they were captured and collared as solitary, territorial wolves (*n* = 2). The intensive study periods included a total of 3198 study days (mean = 39, range = 8–84) where all potential feeding sites were inspected in the field and any carcasses found were classified according to cause and time of death.

During the intensive studies, GPS‐collars were programmed to take a location every half hour (*n* = 30) or every hour (*n* = 52). Wolves were assumed to spend time at, or in the vicinity of, carcasses, in order to handle, consume, and digest the food. All locations within 200 m from one another (i.e., a cluster) were visited in the field after the GPS‐collared wolves had left the area and searched for carcasses, with the aid of dogs during summer. Field crew searched for carcasses within a 100 m radius of clustered GPS‐locations for the entire study period (Sand et al., 2005). In addition, single locations were occasionally visited and searched for carcasses.

For all carcasses found, field crew identified the species and classified the cause of death as either wolf predation or dead by other cause (Sand et al., 2005, 2008). In this study, wolf‐killed carcasses included (1) “fresh wolf‐killed ungulates,” that were classified as killed by wolves based on signs of hunting tracks and/or heavy bleeding/fresh blood at carcass site and if the estimated time of death of the animal coincided with the time of the first GPS‐location of the collared wolf, (2) “old wolf‐killed ungulates,” based on previously mentioned signs of wolf‐kills when time of death of the carcass was estimated before the study period, (3) “small prey species,” when a non‐ungulate prey species was utilized by wolves, (4) “carnivore prey,” including wolf‐killed wolves, red foxes and domestic dogs, and (5) “livestock,” including domestic ungulates killed by wolves. Scavenged carrion included (1) “other cause of death,” including ungulates that had died from starvation, drowning, disease, or had been killed by another species than wolf, and (2) “anthropogenic origin,” when the cause of death was either linked to human activity, including vehicle collisions, carrion left after hunter harvest, or illegal dumping of livestock carrion. Finally, carcasses that were not possible to classify as either wolf‐killed, other cause of death, or anthropogenic origin were classified as “unknown cause of death.” As this category could be either killed or scavenged by wolves, we calculated both a minimum scavenging estimate by assuming the unknowns to be killed by wolves, and a maximum scavenging estimate by assuming the unknowns died from other reason and were scavenged by wolves. In the unknown category, 54% of the carcasses were estimated to have died before the start of the study periods.

### 2.4 Consumption time

To define wolf consumption time per carcass, we created space–time clusters, which are a set of locations where each location was ≤200 m from the next sequential location, and where ≥1 location within the cluster was within 200 m of a carcass (Carricondo‐Sanchez et al., 2020; Tallian et al., 2022). Here, the dataset was subsampled to hourly locations for equal comparison across studies. Clusters were generated in R (R Core Team, 2021). For each study period, we calculated total consumption time as the number of locations within space–time clusters associated with a carcass. Each feeding location was further classified as either predation or scavenging, depending on cause of death of the carcass. Note that these space–time clusters created on the full dataset may differ from the cluster created continuously during fieldwork. So when a space–time cluster overlapped several carcass sites and at least one of the carcasses was classified as a wolf kill, the cluster was assigned as predation (assuming wolves were there due to their own kill). If the wolves' first visit to the different carcass sites was done at different occasions (*n* = 91), then the cluster was assigned to the carcass that wolves visited most recently in time (assuming wolves were there due to the freshest carcass).

A total of 69,616 GPS‐locations (representing all wolf time) were collected during the intensive studies, of which 14,205 locations were within space–time clusters (defined as consumption time). The space–time clusters consisted of 12,137 locations at wolf kills, 823 locations at scavenging sites, and 1245 locations at carcasses with unknown cause of death. The average total consumption time (number of feeding locations/total number of locations for each study period) for wolves in pack was 22% (range 6–50) and 16% for solitary wolves (range 3–44). The average total consumption time in winter was 22% (range 10–37), 19% in summer (range 3–50), and 20% in autumn (range 6–38).

### 2.5 Moose density

The relative density of moose (per km^2^) was estimated in the areas utilized by wolves during the winter studies using fecal pellet group counts conducted during spring, after snow melt. Counts were conducted on circular sample plots with an area of 100 m^2^.The distribution of sampling plots throughout the wolf territories followed one of two designs, with the predominant being 40 plots distributed evenly along the sides of a square of 1 km^2^, with 100 m distance between plots. The sampling squares were regularly distributed across the wolf territory (38–130 squares per territory). The alternative sampling design consisted of clusters of five plots, arranged along the edges and in the centre of a square of 50 × 50 m. The sample plot clusters were distributed regularly across the wolf territories (38–121 clusters per territory). We counted all pellet groups deposited between leaf fall (October 10) and time of spring count right after snow melt. Due to the cold climate during this time of the year, with temperatures mostly below zero and snow cover, we considered decay of fecal pellet groups as negligible. Pellet counts were converted into moose winter densities by accounting for moose defecation rate (14 pellet groups per day; Rönnegård et al., 2008) and time span between leaf fall and date of count (Sand et al., 2016). Average moose density per square was interpolated using inverse distance weighting in ArcGIS by including the 12 closest squares to any raster cell of 100 m cell size in the wolf territory. For each intensive study, mean moose density was estimated as the mean of all cells falling into an 18 km radius buffer around the centroid of the wolf territory, representing an average wolf territory size (Mattisson et al., 2013). The centroid was located during the annual monitoring of wolves (Åkesson, Svensson, et al., 2022). We used the centroid from the following monitoring season if the same adult wolf pair was still present in the territory, thus accounting for a possible change in area use due to changes in pack composition after reproduction in spring and dispersal of older pups. The centroid from the preceding monitoring season was used if the pair was not present in the territory the following season.

### 2.6 Brown bear density

A relative index of brown bear density was calculated using official statistics on the annual number and spatial locations of harvested brown bears (https://www.rovbase.no), a method shown to reflect bear density and distribution (Kindberg et al., 2009). The relative index of density (per km^2^) was estimated using kernel density estimation in QGIS 3.16.16 with a search radius of 100 km. For each summer study for packs, the mean relative index of brown bear density was estimated within an 18 km radius buffer around the centroid of the wolf territory using the same methods as described for moose density.

### 2.7 Human density

The number of inhabitants (per km^2^) was calculated based on human population size at the municipality level from Sweden (https://www.scb.se/) and Norway (https://www.ssb.no/) and estimated for each intensive study as mean human density within an 18 km radius buffer around the centroid of the wolf territory. The mean human density between the present and the following year of the study was used. This was done to better coincide with the timing of the monitoring period that overlaps two calendar years. The buffers overlapped with several municipalities (mean 4, range 2–7), and the mean human density was calculated. Because solitary wolves were not included in the Scandinavian monitoring system, we lacked official locations of centroids of their territories and instead we used centroids that were extracted from the solitary wolf locations during the study.

### 2.8 Statistical analyses

To analyze variation in the proportion of time spent scavenging versus consuming wolf‐killed prey, we fitted generalized linear mixed models (GLMMs) with a binominal distribution using the R‐package glmmTMB (Brooks et al., 2017). The dependent variable “proportion of time spent scavenging” was defined as the number of locations within space–time clusters assigned to scavenged food divided by total consumption time (i.e., the total number of feeding locations within space–time clusters) per intensive study period. The total consumption time was also included as a weight to account for unequal sample size across studies. We tested with both the maximum and the minimum estimates of scavenging time as dependent variables (results for models using the minimum estimate are shown in the Appendix 1). Wolf ID (either as a pair ID for the adult wolves in a territory or as an individual ID for solitary wolves) was included as a random factor to account for repeated observations from the same wolves. Human density was log‐transformed, and all explanatory continuous variables were centred and standardized, using the *scale* command in R, to improve interpretability of regression coefficients (Schielzeth, 2010). Explanatory variables included in the same models were assessed for multicollinearity using VIF. There were no indications of multicollinearity (VIF < 1.4).

We first analyzed the full dataset using season (summer, autumn, winter), social affiliation of wolves (solitary, pack), and human density (range: 0.82–79.02/km^2^) as explanatory variables. To be able to include seasonal explicit variables, we conducted separate analyses for winter and summer for packs only; the sample size from autumn was too small and seasonal explicit variables were not available for solitary wolves. In the seasonal models, we included the variables inbreeding coefficient (range: *F*_average_ 0.13–0.31, *F*_male_ 0–0.36), brown bear density in summer (as they hibernate in winter, range 0–0.0043/km^2^), and moose density in winter (range 0.25–3.29/km^2^), in addition to human density both in summer and winter.

We used AIC model selection corrected for small sample sizes (AICc) to compare the performance of *F*_average_ and *F*_male_ and retained the one with lowest AICc, when comparing the univariate models, for further analyses. We performed AICc model selection on all combinations of explanatory variables, including an intercept only model. We used Nakagawa's R2 (Lüdecke et al., 2021) to calculate the variance explained by the explanatory variables (marginal) as well as for explanatory variables and the random factors (conditional). We considered models within ΔAICc ≤2 (referred to as top models) to be equally important (Burnham & Anderson, 2002), and conducted the statistical analyses in R (R Core Team, 2021). We calculated 95% CI of parameter estimates with the function confint() from the *stats* R package.

## 3 RESULTS

**FIGURE 2:**
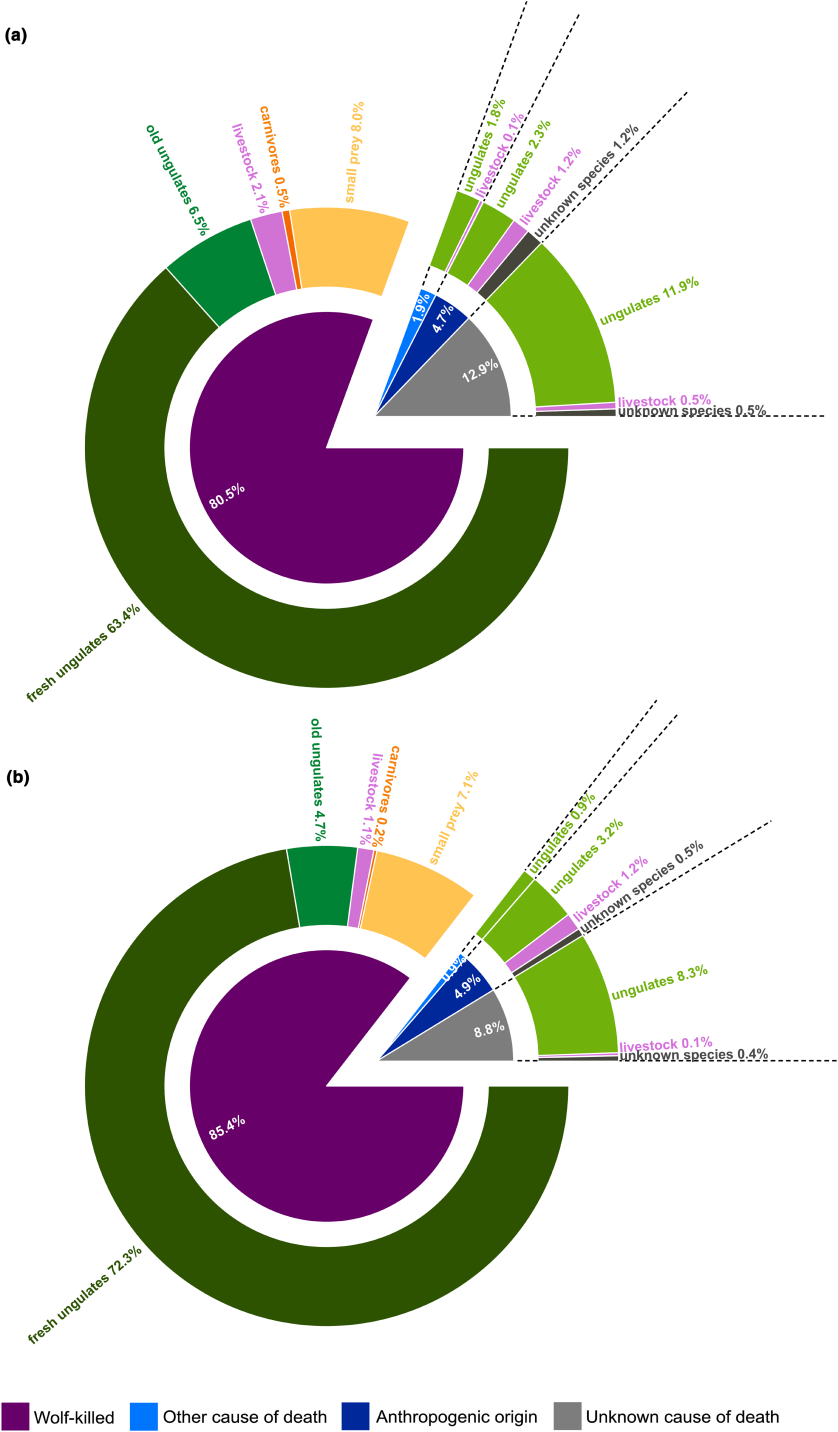
Relative distribution of (a) number of food sources visited by wolves (*n* = 1362) and (b) consumption time (*n* = 14,205 GPS‐locations), during intensive studies of predation (*n* = 82) conducted using GPS‐locations from 39 wolves either solitary (*n* = 11) or in packs (≥2 individuals, *n* = 71) in Scandinavia, 2001–2019. The inner circle shows the cause of death (wolf‐killed, other cause of death, anthropogenic origin, or unknown cause of death) and the outer circle shows wolf‐kills grouped as wild ungulates killed within (fresh) or before the study period (old), livestock, carnivores, or small prey species, while scavenged food sources were grouped as either wild ungulates, livestock, or unknown species.

The intensive studies (*n* = 82) included 1362 observations of wolves utilizing carcasses (in total 1426 of which 64 double or multiple carcasses). Most carcasses were wolf‐killed (80.5%) while a small part had died from other natural causes (1.9%). The remaining had either anthropogenic mortality causes (4.7%), or the cause of death was unknown (12.9%). Ungulates were the most common carcasses (in total 85.9% of which 69.9% were wolf‐killed, Figure 2a, Table 1). The main part of the remaining carcasses consisted of small prey species (8.0%), depredation events (2.1%), scavenging on livestock (1.8%), and unknown species (1.7%). Intraguild predation and intraspecific killing was rare (0.5%) with four out of the seven carcasses almost entirely consumed by wolves.

**TABLE 1:** Carcasses (*n* = 1362) utilized by wolves during 82 intensive studies of predation in Scandinavia during 2001–2019.

Common name	Scientific name	Wolf‐killed^a^	Other cause of death	Anthropogenic origin	Unknown cause of death
Badger	*Meles meles*	18 (1/13/4)			
Beaver	*Castor fiber*	18 (8/10/0)			
Black grouse	*Tetrao tetrix*	25 (11/13/1)			
Capercaillie	*Tetrao urogallus*	11 (5/5/1)			
Cattle	*Bos taurus*	2 (0/2/0)		8 (6/2/0)	
Hooded crow	*Corvus cornix*	1 (1/0/0)			
Dog	*Canis familiaris*	1 (0/0/1)			
Hare	*Lepus* spp.	12 (6/6/0)			
Hazel grouse	*Tetrastes bonasia*	2 (0/2/0)			
Magpie	*Pica pica*	1 (0/1/0)			
Moose	*Alces alces*	818 (420/358/40)	15 (7/7/1)	28 (19/4/5)	122 (81/32/9)
Pig	*Sus scrofa domesticus*			4 (2/1/1)	
Red deer	*Cervus elaphus*	6 (6/0/0)			1 (1/0/0)
Red fox	*Vulpes vulpes*	4 (2/2/0)			
Reindeer	*Rangifer tarandus*	22 (11/11/0)	1 (0/1/0)		5 (5/0/0)
Roe deer	*Capreolus capreolus*	124 (107/17/0)	10 (7/2/1)	3 (3/0/0)	39 (39/0/0)
Sheep	*Ovis aries*	6 (0/6/0)		5 (2/1/2)	1 (0/1/0)
Siberian jay	*Perisoreus infaustus*	1 (0/1/0)			
Squirrel	*Sciurus vulgaris*	1 (1/0/0)			
Unknown bird species	NA	14 (2/12/0)			
Unknown species	NA	2 (0/2/0)		16 (8/1/7)	7 (2/5/0)
Vole	*Cricetidae* spp.	3 (0/3/0)			
Wild boar	*Sus scrofa*	3 (1/2/0)			
Wolf	*Canis lupus*	2 (2/0/0)			

*Note*: Species are specified in alphabetical order according to common name, scientific name, and are grouped as wolf‐killed, other cause of death, anthropogenic origin, or unknown cause of death. Numbers per season are shown in parenthesis according to (winter/summer/autumn).

^a^ All non‐ungulate prey species are assumed to be wolf‐killed.

The main part of wolf consumption time was spent on fresh wolf‐killed ungulates (72.3%, Figure 2b). Wolves spent between 6% (mean, 95% CI: 3–9) and 13% (95% CI: 9–18) of their consumption time scavenging, considering the minimum and maximum estimates, respectively.

### 3.1 Effects of season, social affiliation, and human density

**FIGURE 3:**
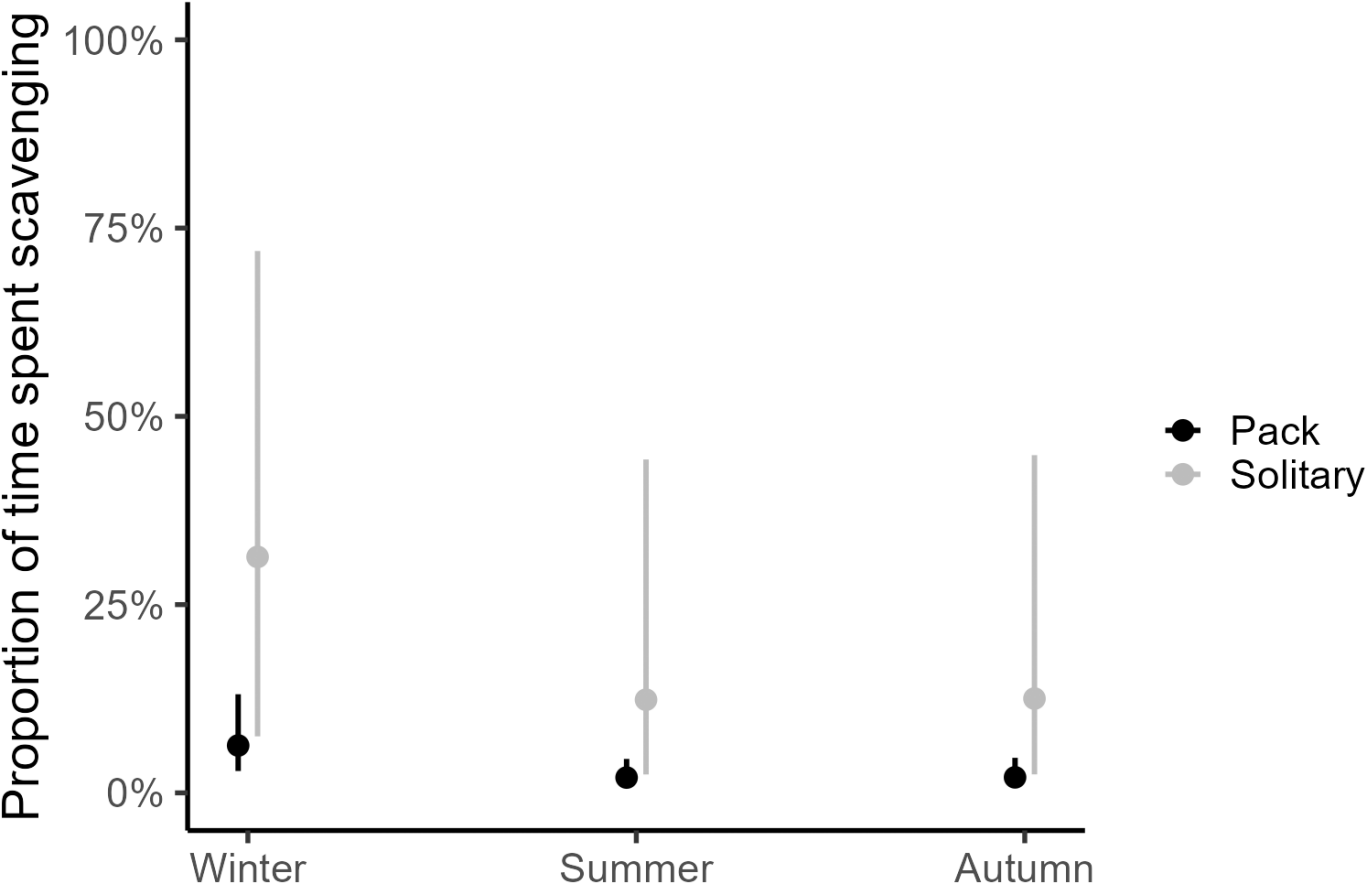
Predicted proportion of maximum estimate of consumption time spent scavenging (±95% CI) in relation to season (winter, summer, autumn) and social affiliation of wolves (pack [black, ≥2 wolves], solitary [gray]) from the highest ranked model based on GPS‐locations of 82 intensive studies of predation in Scandinavia, 2001–2019.

**FIGURE A1:**
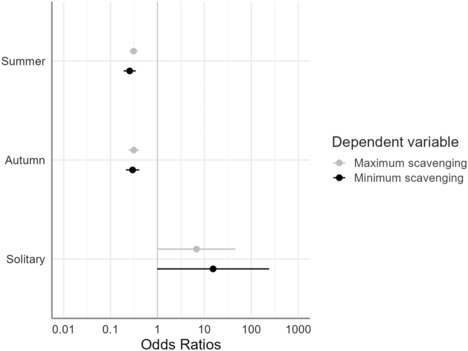
Model output using maximum estimate of the proportion of consumption time spent scavenging (Maximum scavenging, ±95% CI) compared to minimum estimate (Minimum scavenging) for the highest ranked model including season (summer, autumn, winter) and social affiliation of wolves (solitary, pack [≥2 wolves]). The reference values are “winter” for season and “pack” for social affiliation. Data collected during intensive studies of predation (*n* = 82) conducted using GPS‐locations from collared wolves (*n* = 39) in Scandinavia, 2001–2019.

When using the full dataset and the maximum estimate of scavenging time, four models had a ΔAICc ≤2. The highest ranked model included season and social affiliation. Season was retained in all four top models, while social affiliation was only included in two of the four (Table 2). The maximum scavenging time was higher during winter compared to summer and autumn. Scavenging time was also higher for solitary wolves than for packs (Figure 3, Table 3). Human density was included in two of the top models (Table 2). The replacement of the parameter social affiliation with human density (model 3) only indicated weak evidence for a negative relationship, as the confidence interval of the estimates of human density included zero (Table 3), while the addition of human density to the top model (model 4) was uninformative (Tables 2 and 3). The top models with minimum and maximum estimates of scavenging time showed similar results (Figure A1), and the two highest ranked models had the same sets of explanatory variables for minimum (Table A1) and maximum estimates of scavenging time (Table 3). Human density was not included among the top models when using the minimum estimate of scavenging time (Table A1).

**TABLE 2:** Generalized linear mixed models to assess the effect of season (summer, autumn, winter), social affiliation (solitary, pack [≥2 wolves]), human density, average inbreeding coefficient of the adult female and male (*F*_average_), and brown bear density, on the proportion of consumption time spent scavenging by wolves in Scandinavia during 2001–2019.

Dataset	No.	Intercept	Season	Social	Human	*F*_average_	Moose	Bear	df	ΔAICc	LogLik	R2_c_	R2_m_
Annual *n* = 82	1	X	X	X		‐	‐	‐	5	0	−785.8	0.63	0.07
	2	X	X			‐	‐	‐	4	1.5	−787.7	0.64	0.04
	3	X	X		X	‐	‐	‐	5	2.0	−786.8	0.66	0.06
	4	X	X	X	X	‐	‐	‐	6	2.0	−785.7	0.64	0.07
		X			X	‐	‐	‐	3	188.3			
		X		X	X	‐	‐	‐	4	189.9			
		X		X		‐	‐	‐	3	190.8			
		X				‐	‐	‐	2	191.6			
Winter *n* = 35	1	X	‐	‐		X	X	‐	4	0	−440.3	0.79	0.20
	2	X	‐	‐	X	X	X	‐	5	0.3	−439.0	0.79	0.28
	3	X	‐	‐			X	‐	3	1.0	−442.0	0.81	0.15
		X	‐	‐	X		X	‐	4	2.4			
		X	‐	‐		X		‐	3	11.5			
		X	‐	‐				‐	2	11.8			
		X	‐	‐	X	X		‐	4	13.8			
		X	‐	‐	X			‐	3	14.2			
Summer *n* = 27	1	X	‐	‐			‐		2	0	−96.1	0.59	‐
	2	X	‐	‐	X		‐		3	0.6	−95.1	0.578	0.07
	3	X	‐	‐	X		‐	X	4	1.2	−94.1	0.57	0.16
	4	X	‐	‐			‐	X	3	1.7	−95.7	0.58	0.03
		X	‐	‐		X	‐		3	2.2			
		X	‐	‐	X	X	‐		4	3.4			
		X	‐	‐	X	X	‐	X	5	4.0			
		X	‐	‐		X	‐	X	4	4.4			

*Note*: Analyses were conducted using maximum estimates of the proportion of consumption time spent scavenging (for minimum estimates see Table A1). For all tested models, degrees of freedom (df), and difference in AICc relative to the highest‐ranked model (ΔAICc) are shown. For models within ΔAICc ≤2, log‐likelihood (LogLik), conditional (R2_c_) and marginal (R2_m_) Nakagawa's R2 are also shown.

**TABLE 3:** Conditional model parameter estimates (*ß*) with standard error (SE) and 95% CI (explanatory variables shown in bold when not overlapping zero) for each explanatory variable retained in the models with ΔAICc ≤2 (Table 2).

Dataset	Model no.	Explanatory variable	*ß*	SE	95% CI
Annual *n* = 82	1	Intercept	−2.70	0.41	−3.51, −1.89
		**Season: summer**	−1.17	0.10	−1.37, −0.98
		**Season: autumn**	−1.16	0.13	−1.42, −0.90
		**Social affiliation: solitary**	1.92	0.97	0.013, 3.82
	2	Intercept	−2.39	0.40	−3.16, −1.61
		**Season: summer**	−1.17	0.10	−1.37, −0.97
		**Season: autumn**	−1.16	0.13	−1.41, −0.90
	3	Intercept	−2.38	0.41	−3.18, −1.59
		**Season: summer**	−1.17	0.10	−1.36, −0.97
		**Season: autumn**	−1.14	0.13	−1.40, −0.89
		Human density	−0.46	0.37	−1.18, 0.25
	4	Intercept	−2.66	0.43	−3.50, −1.83
		**Season: summer**	−1.17	0.10	−1.37, −0.97
		**Season: autumn**	−1.15	0.13	−1.41, −0.89
		Social affiliation: solitary	1.69	1.07	−0.41, 3.80
		Human density	−0.20	0.38	−0.94, 0.54
Winter *n* = 35	1	Intercept	−3.59	0.70	−4.97, −2.22
		**Moose density**	1.58	0.52	0.56, 2.60
		*F*_average_	1.27	0.66	−0.02, 2.56
	2	Intercept	−3.83	0.70	−5.19, −2.47
		**Moose density**	1.73	0.55	0.64, 2.82
		***F***_**average**_	1.42	0.63	0.19, 2.66
		Human density	1.05	0.66	−0.25, 2.36
	3	Intercept	−3.63	0.78	−5.15, −2.10
		**Moose density**	1.60	0.54	0.54, 2.66
Summer *n* = 27	1	Intercept	−4.14	0.58	−5.27, −3.00
	2	Intercept	−4.14	0.54	−5.21, −3.07
		Human density	0.80	0.56	−0.30, 1.90
	3	Intercept	−4.07	0.50	−5.04, −3.10
		Bear density	0.70	0.46	−0.20, 1.60
		Human density	1.02	0.54	−0.04, 2.07
	4	Intercept	−4.09	0.56	−5.185, −3.00
		Bear density	0.47	0.50	−0.52, 1.46

*Note*: The reference in the analyses is “winter” for season, and “pack” for social affiliation. Analyses were conducted using maximum estimates of the proportion of consumption time spent scavenging for annual, winter and summer intensive studies of wolf predation in Scandinavia, 2001–2019 (for minimum estimate see Table A2).

Individual wolf or pack was responsible for considerable variance in the data in all models shown by higher conditional than marginal R2 values (also in the seasonal models, Tables 2 and A1).

### 3.2 Effects of inbreeding, moose density, and human density during winter

**FIGURE 4:**
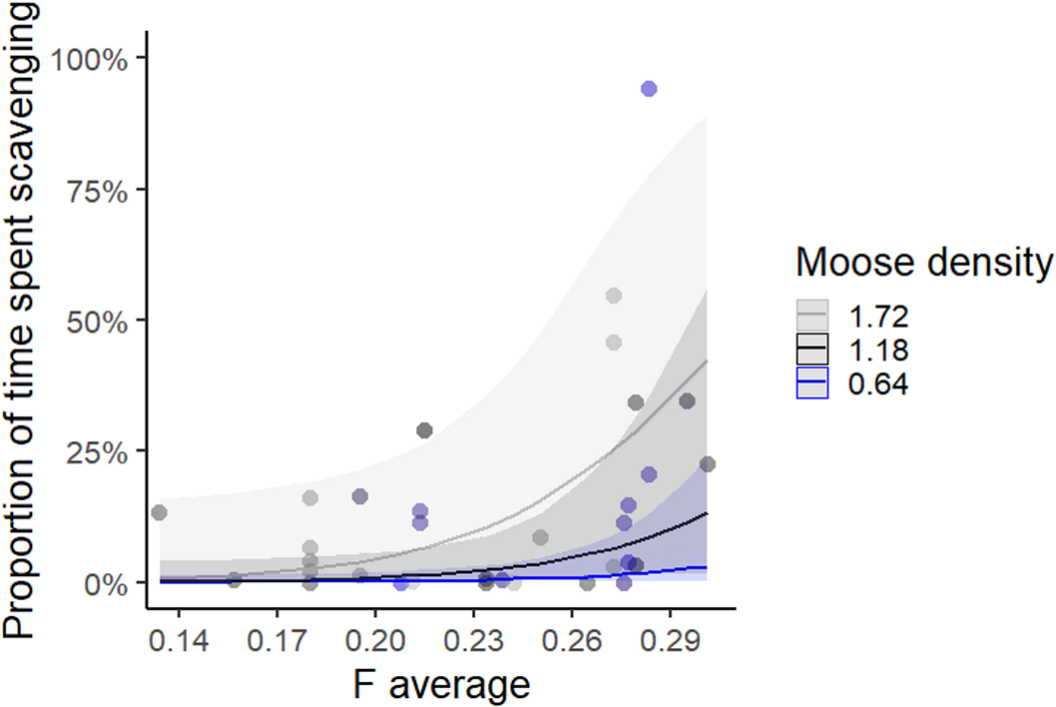
Predicted proportion of maximum estimate of consumption time spent scavenging during winter (±95% CI, unscaled data) in relation to the average inbreeding coefficients of the adult female and male (*F*_average_) and moose density (held constant at three different densities) for the highest ranked model. Dots represent each study period. Data was collected during intensive studies of predation (15 December to 14 May, *n* = 35) for wolves in packs (≥2 wolves) using GPS‐locations from collared wolves (*n* = 23) in Scandinavia, 2001–2019.

**FIGURE A2:**
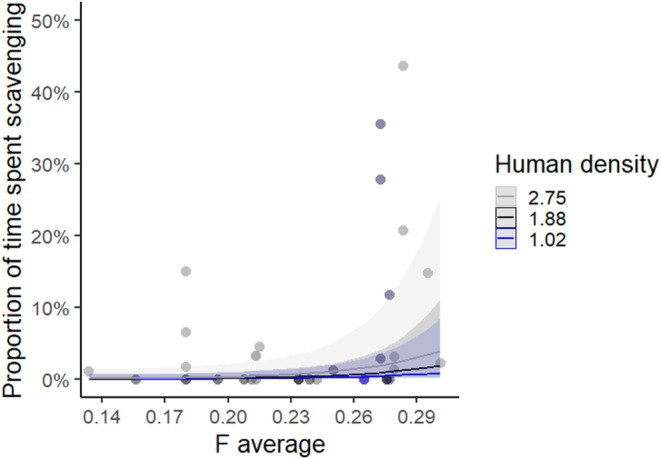
Predicted proportion of minimum estimate of consumption time spent scavenging during winter (±95% CI, unscaled data) in relation to the average inbreeding coefficients of the adult female and male (*F*_average_) and human density (log‐transformed, held constant at three different values) for the second ranked model (ΔAICc = 0.9). Dots represent each study period. Data collected during intensive studies of predation (15 December to 14 May, *n* = 35) for wolves in packs (≥2 wolves) using GPS‐locations from collared wolves (*n* = 23) in Scandinavia, 2001–2019.

When using the winter data and the maximum estimate of scavenging time, three models had a ΔAICc ≤2. The highest ranked model included inbreeding and moose density, with inbreeding retained in two of the three top models while moose density was retained in all (Table 2). Scavenging time increased with both moose density and the inbreeding coefficient *F*_average_ (*F*_average_ performed better [ΔAICc = 1.8] in the AICc model set than *F*_male_, Figure 4, Table 3). Human density was additionally included in the second highest ranked model, with a positive relationship with the maximum estimate of scavenging time (Table 3). However, when using the minimum estimate of scavenging time, moose density was not included among the top models, resulting in inconsistent effects of moose density. The three top models using the minimum estimate of scavenging time included inbreeding (*F*_average_) and human density, as well as the intercept only model (Table A1). Both inbreeding and human density were positively correlated with scavenging time (Table A2, Figure A2), but showed only weak evidence as the intercept only model was included among the top three models.

### 3.3 Effects of inbreeding, brown bear density, and human density during summer

**FIGURE 5:**
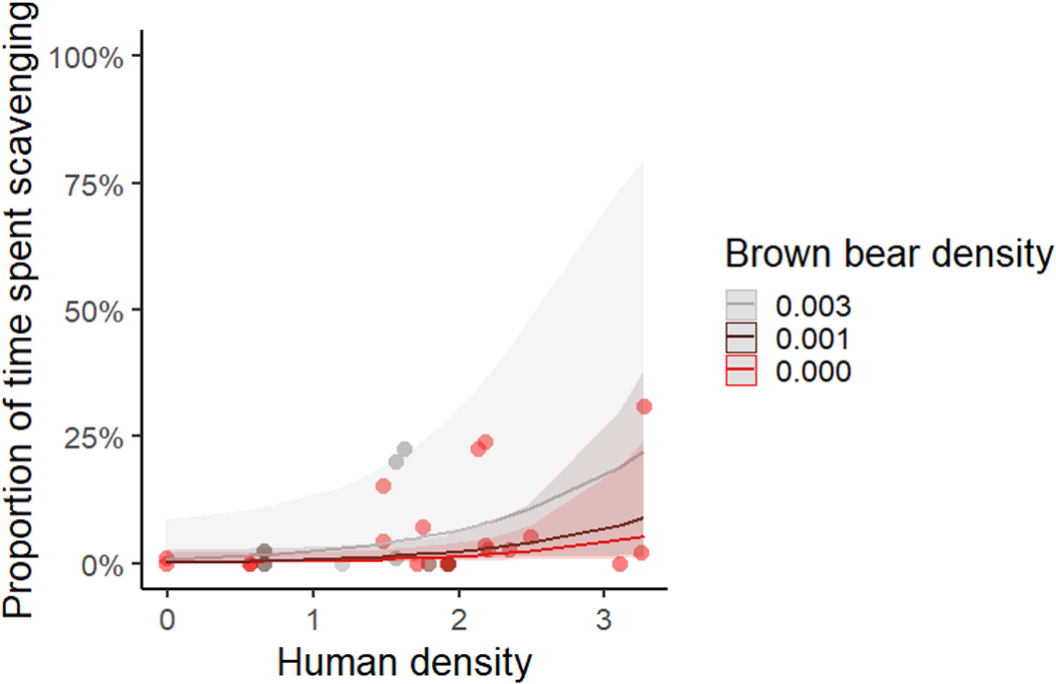
Predicted proportion of maximum estimate of consumption time spent scavenging during summer (±95% CI, unscaled data) in relation to human density (log‐transformed) and brown bear density (held constant at three different densities) for the third ranked model (ΔAICc = 1.2). Dots represent each study period. Data was collected during intensive studies of predation (15 May to 14 September, *n* = 27) for wolves in packs (≥2 wolves) using GPS‐locations from collared wolves (*n* = 21) in Scandinavia, 2001–2019.

When using the summer data and the maximum estimate of scavenging time, four models had a ΔAICc ≤2. The highest ranked model for the summer dataset using the maximum estimate of scavenging time was the intercept only model, and models including brown bear density and human density were each retained in two of the four top models (Table 2). Scavenging time tended to increase with brown bear density and human density (Table 3, Figure 5), but only showed weak evidence as the intercept only model was ranked as the top model and the confidence intervals for the parameter overlapped zero.

**FIGURE A3:**
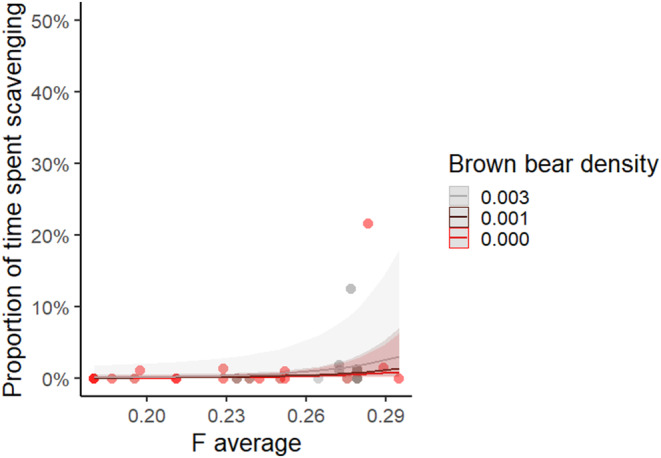
Predicted proportion of minimum estimate of consumption time spent scavenging during summer (±95% CI, unscaled data) in relation to the average inbreeding coefficients of the adult female and male (*F*_average_) and brown bear density (held constant at three different densities) for the second ranked model (ΔAICc = 1.4). Dots represent each study period. Data collected during intensive studies of predation (15 May to 14 September, *n* = 27) for wolves in packs (≥2 wolves) using GPS‐locations from collared wolves (*n* = 21) in Scandinavia, 2001–2019.

The highest ranked model using the minimum estimate of scavenging time included the inbreeding coefficient *F*_average_ only (*F*_average_ performed better [ΔAICc = 2.8] in the AICc model set than *F*_male_), and the model including inbreeding and brown bear density was the second highest ranked model (Table A1) among the two top models. Scavenging time increased with both inbreeding and brown bear density (Table A2, Figure A3).

## 4 DISCUSSION

Our results suggest that the extent of scavenging varied among individuals, and that solitary and inbred wolves in generally devoted more time to scavenging. Extrinsic factors such as the density of main prey species, intraguild competitors, and humans also affected wolves propensity to scavenge. However, despite the extreme inbreeding levels among Scandinavian wolves (Åkesson et al., 2016), and humans seasonally providing large amounts of biomass from hunter harvest remains (Wikenros et al., 2013), wolves in Scandinavia mainly consumed wolf‐killed ungulates. The lack of strong evidence for several of the explanatory variables is likely because scavenging time overall was low for wolves in Scandinavia compared to time spent on their own kills.

Despite a 10 times greater availability of biomass from moose carrion in autumn, mainly consisting of remains from hunter harvested moose (Wikenros et al., 2013), scavenging time was not greater in autumn. Remains from hunting included both internal organs and rumen left in the forest after a moose was shot and dumps of slaughter remains (mainly bones). In a previous study conducted during autumn, wolves did not turn up on camera‐monitored hunter harvest remains inside wolf territories (Wikenros et al., 2013), supporting the results from this study. It is likely that wolves avoid scavenging on remains from hunter harvest in autumn due to the pulse in human hunting activity during that time. Such avoidance could also be expected when considering that the mortality of wolves is largely due to anthropogenic factors (legal culling, verified and cryptic poaching, and vehicle collisions) and to a lesser degree to natural causes of death (Liberg et al., 2020). The majority of moose are harvested during October, although the harvest continues at a lower intensity until the end of February (Wikenros et al., 2013). The increased scavenging time during winter may be due to less activity by hunters in the forest compared to autumn. Wolves in Scandinavia are also known to avoid human settlements and main roads (Carricondo‐Sanchez et al., 2020; Zimmermann et al., 2014), further supporting the idea that wolves may avoid areas with high human activity.

Biomass from vehicle collisions and other causes of death (starvation etc.) constituted a smaller part (7% and 10%, respectively) of available carcass biomass within wolf territories as compared to remains from hunter harvest of moose (57%) (Wikenros et al., 2013). However, biomass from vehicle collisions is higher in winter than in summer, and with less variation in availability throughout the year, compared to remains from hunter harvest which are generally available in short pulse (Wikenros et al., 2013). Two factors may help explain greater wolf scavenging time during winter. First, the deaths caused by starvation, despite to a low extent, occurs in late winter when the body condition of moose is known to be at its lowest (Cederlund et al., 1991; Sand et al., 2012). Second, cold temperatures during winter keep carcasses fresher and make them last longer, increasing availability for scavenging, compared to the warmer summer period.

Our prediction that solitary wolves scavenge more than packs was confirmed. We were not able to separate the different age classes of solitary wolves in the analyses given the small sample size. This, and the large variation in scavenging time among solitary wolves, makes it difficult to draw clear conclusions. However, the observed variation may reflect the diversity among solitary wolves that recently left their natal territory and dispersed through an unknown landscape, as compared to older and more experienced solitary wolves that may suffer from reduced efficiency compared to pack hunting (Sand et al., 2006a; Zimmermann et al., 2015). There is evidence that larger and more experienced wolves in Scandinavia have greater hunting success (Sand et al., 2006a), which is in line with findings from other systems showing an effect of sex, age, and body size on hunting success (MacNulty, Smith, Mech, et al., 2009; MacNulty, Smith, Vucetich, et al., 2009). Packs led by older males were more successful at hunting moose than packs led by younger males, and the hunting success of packs was more dependent on male age than on female age, with males being 25%–30% larger than females (Sand et al., 2006a). The observed variation in scavenging time among wolves in packs may also be explained by age and experience as well as body condition and pack size. For wolves living in large packs, the level of scavenging may increase. This is because large packs often kill fewer ungulates than required to cover the field metabolic rate of all pack members, especially at low moose abundance (Zimmermann et al., 2015). In contrast, small packs experience less intraspecific competition for biomass of killed prey and may therefore rely less on scavenging.

The level of inbreeding in the wolves affected individual foraging behavior, especially during winter. Inbreeding is expected to negatively affect body condition (Laikre & Ryman, 1991) that in turn may affect hunting success, leading to increased consumption time of more easily accessed carrion. Scavenging time increased in areas with high moose density and highly inbred wolves. Unfortunately, sample sizes were too small to test for an interaction between moose density and inbreeding. We predicted that wolves in low moose density areas would increase scavenging time as they would have to devote more of their time for finding vulnerable prey (Zimmermann et al., 2015). In contrast, our results showed the opposite pattern. This may have been caused by an increased availability of remains from hunter harvest at high moose densities that wolves, maybe especially inbred ones, could utilize, but this needs to be further investigated.

We found weak evidence that scavenging time increased with brown bear density during summer, as would be expected due to exploitation competition (Tallian et al., 2022). Both brown bears and wolves prey heavily on neonate moose during summer in Scandinavia (Ordiz et al., 2020) and brown bear predation on neonates is generally expected to be additive to wolf predation (Griffin et al., 2011). Furthermore, wolves in Scandinavia prey primarily on newly born moose calves during this time, only occasionally hunting the less vulnerable adult and subadult age classes. Together, wolves and brown bears deplete the supply of shared neonate prey on the landscape, decreasing the overall seasonal density of their main prey. Thus, there are likely fewer vulnerable prey on the landscape in areas where brown bear density is high, which may facilitate a shift toward wolf scavenging (Tallian et al., 2022).

Scavenging time during summer and winter increased with human density in line with our prediction, although only with weak evidence. This likely reflects that the scavenging behavior of wolves, and/or availability of human‐provided carrion, may be influenced not only by human density itself but also by human activities in the landscape. In addition, human density may not always be a straightforward index of human activity. Most moose hunting occurs in remote areas, resulting in seasonally high availability of biomass to scavenge in low human density areas, while other types of anthropogenic food sources are likely more predictable in time and available in areas with high human density. Since our response variable (scavenging time) was calculated at the territory level, we also included human density at the territory level. Therefore, our analyses do not reflect how humans may affect wolf feeding behavior at specific carcasses nor capture the temporal variation and spatial heterogeneity in carcass availability due to humans but show the overall effect of human density on scavenging time at the territory level.

Consumption of other carnivores within the same guild is usually rare. However, Martins et al. (2020) documented increased carnivore–carnivore consumption in human‐dominated landscapes with higher densities of mesopredators and lower availability of wild and domestic prey species. Different hypotheses have been suggested as to why carnivores kill other carnivores, that is, food acquisition, competition, aggressive behavior (Martins et al., 2020). The generally high moose densities in Scandinavia make it unlikely that food acquisition was the reason behind the occasional intraguild predation events, despite the fact carcasses were partly consumed by wolves. Intraspecific killing in the Scandinavian wolf population was low, with only two wolf carcasses found during the study period (assuming that the remains of killed wolves would be found with the same methodology used for finding other carcasses). Infrequent intraspecific aggression has been reported also in other lower density wolf populations contrary to what has been observed in denser wolf populations where intraspecific strife is the main natural cause of death (Mech & Boitani, 2003).

The low utilization by wolf packs of human‐provided carrion, in combination with a low incidence of livestock depredations, contrasts with other anthropogenic landscapes where depredation by wolves is high (Vos, 2000), or where humans provide carcass dumps and garbage sites that are heavily utilized by wolves (Ciucci et al., 2020; Newsome et al., 2015). High levels of depredation and use of anthropogenic food sources can increase conflicts over carnivores and their possible impacts on human livelihoods (Newsome et al., 2015). The foraging pattern dominated by predation on wild prey species in Scandinavia may in this respect contribute to lower levels of conflict. However, in Scandinavia, and elsewhere in Europe, humans control densities of ungulates to a large extent via hunter harvest (Jensen et al., 2020; Linnell et al., 2020). In addition, humans contribute to increased wolf hunting success of moose in Scandinavia. This is likely because harvest is the main mortality factor for moose, even within wolf territories, and possibly also because the mode of hunting moose with baying dogs affects moose behavior making them predator‐naïve (Sand et al., 2006b).

When wolves primarily hunt, rather than scavenge, wolf predation can have a large impact of the possible harvest yield of important game species (Wikenros et al., 2015, 2020). This represents another source of conflict in landscapes where humans, and not large carnivores, are the main mortality factor in ungulate populations. In Scandinavia, the anthropogenic impact likely affects wolf feeding behavior through avoidance of human activities (Carricondo‐Sanchez et al., 2020) resulting from the fact that humans, historically have been, and still are, the main source of mortality in the wolf population (Liberg et al., 2020; Wabakken et al., 2001). In contrast to seasonal pulsed harvest, wolf predation provides a year around access to scavenging opportunities for other species, both in Scandinavia (Wikenros et al., 2013) and elsewhere (Wilmers et al., 2003; Wilmers & Getz, 2005). In addition, the low wolf scavenging rate observed in Scandinavia means that co‐occurring scavenger species will not have dramatically reduced access to carrion if wolves re‐colonize their area.

## AUTHOR CONTRIBUTIONS

**Camilla Wikenros:** Conceptualization (lead); data curation (lead); formal analysis (lead); funding acquisition (equal); investigation (lead); methodology (lead); project administration (lead); visualization (equal); writing – original draft (lead); writing – review and editing (lead). **Cecilia Di Bernardi:** Conceptualization (equal); data curation (lead); formal analysis (equal); investigation (equal); methodology (equal); writing – review and editing (equal). **Barbara Zimmermann:** Conceptualization (equal); data curation (equal); formal analysis (equal); funding acquisition (equal); methodology (equal); writing – review and editing (equal). **Mikael Åkesson:** Data curation (equal); writing – review and editing (equal). **Maike Demski:** Conceptualization (equal); data curation (equal); writing – review and editing (supporting). **Øystein Flagstad:** Data curation (equal); writing – review and editing (equal). **Jenny Mattisson:** Conceptualization (equal); formal analysis (equal); writing – review and editing (equal). **Aimee Tallian:** Conceptualization (equal); writing – review and editing (equal). **Petter Wabakken:** Data curation (supporting); funding acquisition (equal); writing – review and editing (equal). **Håkan Sand:** Conceptualization (equal); data curation (equal); funding acquisition (equal); writing – review and editing (equal).

## FUNDING INFORMATION

This study was funded by the Swedish Research Council FORMAS (2019‐01186), Swedish Environmental Protection Agency, Norwegian Environment Agency, and Interreg Sweden‐Norway.

## Data Availability

Data and R script available from the Dryad Digital Repository: https://doi.org/10.5061/dryad.80gb5mktr.

## APPENDIX 1

**TABLE A1:** Generalized linear mixed models to assess the effect of season (summer, autumn, winter), social affiliation (solitary, pack [≥2 wolves]), human density, average inbreeding coefficient of the adult female and male (*F*_average_), and brown bear density on the proportion of consumption time spent scavenging of wolves in Scandinavia during 2001–2019.

Dataset	No.	Intercept	Season	Social	Human	*F*_average_	Moose	Bear	df	ΔAICc	LogLik	R2_c_	R2_m_
Annual *n* = 82	1	X	X	X		‐	‐	‐	5	0	−397.8	0.76	0.08
	2	X	X			‐	‐	‐	4	1.3	−399.6	0.78	0.03
		X	X	X	X	‐	‐	‐	6	2.3			
		X	X		X	‐	‐	‐	5	2.8			
		X		X		‐	‐	‐	3	124.1			
		X				‐	‐	‐	2	125.0			
		X			X	‐	‐	‐	3	125.3			
		X		X	X	‐	‐	‐	4	125.8			
Winter *n* = 35	1	X	‐	‐		X		‐	3	0	−227.8	0.71	0.11
	2	X	‐	‐	X	X		‐	4	0.9	−227.0	0.72	0.18
	3	X	‐	‐				‐	2	0.9	−229.5	0.72	
		X	‐	‐		X	X	‐	4	2.1			
		X	‐	‐	X			‐	3	2.4			
		X	‐	‐	X	X	X	‐	5	2.8			
		X	‐	‐			X	‐	3	3.2			
		X	‐	‐	X		X	‐	4	4.8			
Summer *n* = 27	1	X	‐	‐		X	‐		3	0	−32.7	0.63	0.27
	2	X	‐	‐		X	‐	X	4	1.4	−32.0	0.63	0.30
		X	‐	‐	X	X	‐		4	2.8			
		X	‐	‐			‐	X	3	2.8			
		X	‐	‐			‐		2	3.2			
		X	‐	‐	X		‐	X	4	3.8			
		X	‐	‐	X	X	‐	X	5	3.9			
		X	‐	‐	X		‐		3	5.3			

*Note*: Analyses were conducted using minimum estimates of the proportion of consumption time spent scavenging. For all tested models, degree of freedom (df), and difference in AICc relative to the highest‐ranked model (ΔAICc) are shown. For models within ΔAICc ≤2, log‐likelihood (LogLik), conditional (R2_c_) and marginal (R2_m_) Nakagawa's R2 are also shown.

**TABLE A2:** Conditional model parameter estimates (*ß*) with standard error (SE) and 95% CI (explanatory variables shown in bold when not overlapping zero) for each explanatory variable retained in the models within ΔAICc ≤2 shown in Table A1.

Dataset	Model no.	Explanatory variable	*ß*	SE	95% CI
Annual *n* = 82	1	Intercept	−5.22	0.68	−6.56, −3.87
		**Season: summer**	−1.36	0.15	−1.66, −1.07
		**Season: autumn**	−1.22	0.17	−1.55, −0.89
		Social affiliation: solitary	2.73	1.40	−0.02, 5.48
	2	Intercept	−4.81	0.66	−6.11, −3.52
		**Season: summer**	−1.36	0.15	−1.65, −1.06
		**Season: autumn**	−1.22	0.17	−1.54, −0.89
Winter *n* = 35	1	Intercept	−5.42	0.77	−6.93, −3.92
		*F*_average_	1.10	0.61	−0.08, 2.30
	2	Intercept	−5.58	0.78	−7.10, −4.06
		***F***_**average**_	1.19	0.60	0.03, 2.35
		Human density	0.76	0.60	−0.42, 1.95
	3	Intercept	−5.47	0.84	−7.11, −3.82
Summer *n* = 27	1	Intercept	−6.26	0.74	−7.70, −4.81
		***F***_**average**_	1.54	0.64	0.29, 2.80
	2	Intercept	−6.22	0.71	−7.60, −4.84
		*F*_average_	0.55	0.48	−0.39, 1.50
		**Bear density**	1.35	0.65	0.08, 2.62

*Note*: The reference in the analyses is “winter” for season, and “pack” for social affiliation. Analyses were conducted using minimum estimates of the proportion of consumption time spent scavenging for annual, winter and summer intensive studies of wolves in Scandinavia, 2001–2019.
